# Effect of Ramadan Fasting on Blood Lipid Profile Among Populations in the South Asia Region: A Systematic Review and Meta-Analysis

**DOI:** 10.7759/cureus.86309

**Published:** 2025-06-18

**Authors:** Zayna Motiwala, Pritul Sarker, Oleksii Hliebov

**Affiliations:** 1 Department of Medical Foundations, Ross University School of Medicine, Bridgetown, BRB

**Keywords:** awareness of cardiovascular disease, blood lipid profile, dietary behavior, ramadan fasting, south asia

## Abstract

Ramadan fasting involves abstinence from food and drink during daylight hours for 30 consecutive days. While beneficial effects of Ramadan intermittent fasting (RIF) on cardiometabolic risk factors have been suggested, findings related to blood lipid profiles remain inconsistent, particularly among South Asians, who are highly predisposed to dyslipidemia and cardiovascular disease. This systematic review and meta-analysis aimed to evaluate the effects of RIF on lipid profiles among selected South Asian populations from India, Pakistan, Bangladesh, Nepal, and Sri Lanka. These countries were selected due to their disproportionately higher cardiovascular risk burden, shared dietary practices, and availability of comparable studies. A comprehensive literature search was conducted according to Preferred Reporting Items for Systematic Reviews and Meta-Analyses (PRISMA) guidelines across PubMed, Embase, Scopus, Web of Science, and Google Scholar up to March 2025, retrieving 2,430 records; 10 studies (n=432) comprising randomized controlled trials and observational cohort studies in adult populations (healthy and those with controlled metabolic conditions) met all inclusion criteria. Meta-analysis using random-effects models demonstrated significant lipid improvements post-Ramadan, including increased high-density lipoprotein cholesterol (HDL-C) (Z=2.50, p=0.01), decreased low-density lipoprotein cholesterol (LDL-C) (Z=2.19, p=0.03), and decreased total cholesterol (TC) (Z=2.11, p=0.03). No statistically significant change was observed for triglycerides (TG) (Z=0.27, p=0.78). Substantial heterogeneity (I^2^ > 80%) was observed for all lipid parameters, likely due to methodological differences, varied dietary adherence, baseline health status, and cultural dietary habits. Variability was explored via sensitivity analyses and qualitative assessment of individual study characteristics. Unlike other lipids, TG responses varied considerably, potentially attributable to increased consumption of fried and fatty foods during the non-fasting hours, reduced lipoprotein lipase activity, and increased mobilization of free fatty acids. These findings indicate that RIF can confer cardiovascular benefits through improved HDL-C, LDL-C, and TC, though TG outcomes remain influenced by dietary and metabolic factors. Future research should incorporate structured dietary guidance during Ramadan and investigate genetic, gender-specific, and lifestyle determinants to reduce heterogeneity and optimize cardiovascular outcomes associated with RIF.

## Introduction and background

Nearly two billion Muslims participate annually in the holy month of Ramadan, practicing Ramadan intermittent fasting (RIF), which involves abstaining from food, drink, and unhealthy habits from sunrise until sunset for approximately 30 consecutive days [1]. This extended daily fasting triggers significant biological changes, such as enhanced lipolysis, altered insulin secretion patterns, increased free fatty acid mobilization, and reduced lipoprotein lipase activity, mechanisms that can influence lipid metabolism, energy balance, and overall metabolic health [2]. South Asian populations represent a substantial portion of the global Muslim population and exhibit a disproportionately higher risk of cardiovascular diseases (CVDs) and metabolic syndrome as compared to Caucasian and African populations, and to immigrants from other Asian groups [3]. This elevated risk profile is attributed to multiple factors, including genetic predisposition to insulin resistance and central adiposity, diets rich in refined carbohydrates and fats, and notably lower physical activity levels compared to other populations [4,5]. Furthermore, substantial variations exist within South Asian countries themselves, with urban populations typically exhibiting greater CVD risks associated with higher consumption of processed foods, sedentary lifestyles, and socioeconomic stressors. In contrast, rural populations face different health challenges influenced by resource constraints and limited healthcare access.

Previous studies on predominantly Middle Eastern populations have suggested that RIF generally improves blood lipid profiles, reporting reductions in total cholesterol (TC), low-density lipoprotein cholesterol (LDL-C), triglycerides (TG), and increases in high-density lipoprotein cholesterol (HDL-C) [2]. However, the dietary and fasting practices among South Asian populations differ considerably from Middle Eastern populations due to cultural practices, climatic factors, and geographical latitude differences affecting fasting duration. For example, South Asian meals during iftar (breaking the fast) frequently involve fried and carbohydrate-rich foods, potentially negating some lipid-lowering benefits observed elsewhere. Additionally, fasting hours vary significantly between Middle Eastern and South Asian regions due to geographical latitude differences, potentially impacting metabolic outcomes [6,7].

Existing research examining the effect of RIF on lipid metabolism specifically among South Asian populations has produced inconsistent findings. While some studies have reported favorable lipid profile improvements, such as reductions in TC and LDL-C [8,9], others have observed neutral or even adverse lipid changes, such as increased TG [10]. Such inconsistencies in reported outcomes may be attributable to several factors, broadly grouped into biological (e.g., age, gender differences, baseline metabolic status, genetic predispositions, and hormonal variations), environmental and cultural (e.g., dietary composition, caloric intake patterns during Ramadan, physical activity levels, and smoking behaviors), and methodological factors (e.g., variations in study designs, timing of lipid profile measurements, participant selection criteria, and sample sizes) [11]. Given this variability and the substantial heterogeneity among study findings, it is essential to systematically synthesize available evidence to clarify the overall impact of RIF on lipid profiles within the selected South Asian populations.

Objectives

The objective of this study was to systematically evaluate and quantify the impact of RIF on blood lipid profiles (TC, LDL-C, HDL-C, TG) among selected South Asian populations (India, Pakistan, Bangladesh, Nepal, and Sri Lanka) and assess the immediate (≤ 7 days post-Ramadan) and short-term (four to eight weeks post Ramadan) sustainability of lipid profile changes observed following RIF.

Hypothesis

RIF is associated with overall favorable lipid changes in South Asian populations, specifically, increased HDL-C and decreased LDL-C and TC, while its impact on TG is expected to be variable and may depend on baseline metabolic and dietary factors.

Research questions

This review addresses the question “How does RIF affect blood-lipid profiles (TC, LDL-C, HDL-C, TG) in South Asian populations (India, Pakistan, Bangladesh, Nepal, Sri Lanka)?” We also aimed to explore various factors that could contribute to the variability observed in findings from studies assessing the relationship between RIF and lipid profiles in these South Asian nations by answering the following sub-questions: “Do lipid outcomes differ by study design or presence versus absence of a non-fasting control group?”, “How do age, sex, baseline metabolic status, and concomitant medication use influence lipid responses to RIF?”, “To what extent do country-specific dietary patterns, meal composition at ifṭār/suḥūr, nutrition-education interventions, physical activity, and smoking cessation during Ramadan alter lipid changes?”, and “Are any observed lipid changes maintained after Ramadan ends?”

## Review

Methodology

This systematic review was conducted and reported according to the Preferred Reporting Items for Systematic Reviews and Meta-Analyses (PRISMA) guidelines [12].

Search Sources and Strategy

A comprehensive, in-depth literature search was conducted in electronic databases, including PubMed, Scopus, Web of Science, and Google Scholar, from inception to March 2025. The search strategy combined Medical Subject Headings (MeSH) and free-text terms using Boolean operators (AND, OR) in three concept clusters: (1) related to Ramadan fasting modalities (“Ramadan fasting", “intermittent fasting", “Islamic fasting", “Ramadan”), (2) changes in lipid profile (“lipid profile", “cholesterol", “triglycerides", “low-density cholesterol", “low-density lipoprotein cholesterol", “high-density cholesterol", “high-density lipoprotein cholesterol", “LDL-C", “HDL-C”), and (3) South Asian populations (“South Asia", “South Asian", “India", “Pakistan", “Bangladesh", “Sri Lanka", “Nepal”, “Afghanistan", “Maldives", “Bhutan”). Within each cluster, synonyms were linked with OR. Subject headings were ‘exploded’ to capture all narrower terms. Detailed syntax for PubMed and translated strategies for other databases are provided in Table 1.

**Table 1:** Search syntax used to identify studies on Ramadan fasting, lipid profile changes, in South Asian populations

Database	Example of the search string
PubMed (MEDLINE)	("Fasting"[MeSH Terms] OR "Ramadan fasting"[Title/Abstract] OR "intermittent fasting"[Title/Abstract] OR "Islamic fasting"[Title/Abstract] OR "Ramadan"[Title/Abstract]) AND ("Lipids"[MeSH Terms] OR "lipid profile"[Title/Abstract] OR "cholesterol"[Title/Abstract] OR "triglycerides"[Title/Abstract] OR "low-density cholesterol"[Title/Abstract] OR "low-density lipoprotein cholesterol"[Title/Abstract] OR "high-density cholesterol"[Title/Abstract] OR "high-density lipoprotein cholesterol"[Title/Abstract] OR "LDL-C"[Title/Abstract] OR "HDL-C"[Title/Abstract]) AND ("South Asia"[Title/Abstract] OR "South Asian"[Title/Abstract] OR "India"[Title/Abstract] OR "Pakistan"[Title/Abstract] OR "Bangladesh"[Title/Abstract] OR "Sri Lanka"[Title/Abstract] OR "Nepal"[Title/Abstract] OR "Afghanistan"[Title/Abstract] OR "Maldives"[Title/Abstract] OR "Bhutan"[Title/Abstract])
Scopus	(TITLE-ABS-KEY("Ramadan fasting" OR "intermittent fasting" OR "Islamic fasting" OR "Ramadan")) AND (TITLE-ABS-KEY("lipid profile" OR "cholesterol" OR "triglycerides" OR "low-density cholesterol" OR "low-density lipoprotein cholesterol" OR "high-density cholesterol" OR "high-density lipoprotein cholesterol" OR "LDL-C" OR "HDL-C")) AND (TITLE-ABS-KEY("South Asia" OR "South Asian" OR "India" OR "Pakistan" OR "Bangladesh" OR "Sri Lanka" OR "Nepal" OR "Afghanistan" OR "Maldives" OR "Bhutan"))
Google Scholar	("Ramadan fasting" OR "intermittent fasting" OR "Islamic fasting" OR Ramadan) AND ("lipid profile" OR cholesterol OR triglycerides OR "LDL-C" OR "HDL-C") AND ("South Asia" OR "South Asian" OR India OR Pakistan OR Bangladesh OR "Sri Lanka" OR Nepal OR Afghanistan OR Maldives OR Bhutan)
Web of Science	TS=("Ramadan fasting" OR "intermittent fasting" OR "Islamic fasting" OR "Ramadan") AND TS=("lipid profile" OR cholesterol OR triglycerides OR "low-density cholesterol" OR "low-density lipoprotein cholesterol" OR "high-density cholesterol" OR "high-density lipoprotein cholesterol" OR "LDL-C" OR "HDL-C") AND TS=("South Asia" OR "South Asian" OR India OR Pakistan OR Bangladesh OR "Sri Lanka" OR Nepal OR Afghanistan OR Maldives OR Bhutan)

References from retrieved articles and relevant reviews were screened manually to identify additional eligible studies using the Rayyan web application for systematic reviews [13]. The search retrieved 2,430 records; after removal of 113 duplicates, 2,317 unique titles/abstracts were screened, 100 full texts were examined in detail, and 10 studies met all inclusion criteria.

Inclusion Criteria

We included studies published in peer-reviewed journals in English with free full-text access that examined the effect of RIF on the blood lipid profile in the South Asian population. No studies from Afghanistan, Bhutan, or the Maldives met the inclusion criteria, so pooled estimates are based on data from Bangladesh, India, Nepal, Pakistan, and Sri Lanka.

Population: Studies conducted on adult individuals (≥18 years) in South Asian populations (individuals living in and originating from India, Pakistan, Bangladesh, Nepal, and Sri Lanka), including healthy individuals and those with controlled chronic diseases (e.g., diabetes, hypertension, dyslipidemia).

Intervention/Exposure: Studies evaluating fasting during the entire month of Ramadan (complete fasting from dawn until sunset during Ramadan).

Comparison: Studies comparing at least one parameter from the lipid profile before and after RIF: TC, LDL-C, HDL-C, TG.

Outcomes: Studies reporting at least one pre-Ramadan and post-Ramadan blood lipid profile measurement: TC, LDL-C, HDL-C, TG.

Study design: Original research articles, including randomized controlled trials, prospective or retrospective cohort studies, and cross-sectional studies.

Exclusion Criteria

The following exclusion criteria were applied to the retrieved articles to eliminate factors that may incur potential quality or methodological issues. Studies were excluded if they met one or more of the following conditions: (1) conducted outside of South Asian populations, or if data specific to South Asians were not separately reported or cannot be separately analyzed; (2) studies that include partial or alternate-day fasting interventions that do not reflect traditional RIF; (3) studies that were conducted on fasting pregnant women, lactating mothers, pediatric (<18 years), or critically ill patients; (4) studies that did not report lipid profile outcomes quantitatively (e.g., studies that expressed changes in blood lipid profile using curves and bar graphs without reporting exact numerical values); (5) studies that involved special physical activity or dietary plans during RIF; (6) unpublished, non-peer-reviewed data; (7) studies that reported the post-Ramadan measurement after two month or longer; (8) studies without full-text availability or data insufficient for meta-analysis; (9) non-original studies (e.g., reviews, letters, editorials, conference abstracts) and (10) studies published in languages other than English.

Screening Process

The principal outcome of this review was to report the effect of RIF on changes in blood lipid profile (TC, LDL-C, HDL-C, TG) in the selected South Asian population. All identified articles were transferred to the online software Rayyan QCRI [13]. Two independent reviewers used Rayyan QCRI online software to screen the titles and abstracts of identified studies for eligibility based on the inclusion and exclusion criteria. They then retrieved the full texts of potentially eligible articles and independently evaluated them for final inclusion. Inter-rater reliability was high (Cohen’s κ = 0.82 for title/abstract screening and κ = 0.87 for full-text eligibility). Any disagreements regarding study selection were resolved through discussion or by consulting a third reviewer.

Data Extraction

Data extraction was performed on the included full-text articles. Initially, the abstracts of each study were reviewed, followed by a full-text evaluation of each study that met the inclusion criteria. Two authors performed data extraction independently using a standardized Excel Spreadsheet (Microsoft Corporation, Redmond, Washington, United States) explicitly created for this review. Extracted data included first author’s name, publication year, country of the study, study design and sample size, participant characteristics (age, gender, health status, baseline lipid levels (TC, LDL-C, HDL-C, TG), ethnicity, country of origin), methods of participant recruitment, fasting duration and dietary practices during Ramadan, pre-Ramadan and post-Ramadan values for blood lipid parameters, statistical measures (means, standard deviations, confidence intervals, p-values), main findings, study limitations, and study outcomes. Discrepancies during data extraction were resolved by consensus or through third-reviewer arbitration.

Where authors reported an exact duration of fasting, we converted the value to hours with one decimal place and then classified it into three a priori categories for qualitative comparison: <14 hours, 14-16 hours, and >16 hours. Narrative descriptions related to the diet were coded dichotomously as either (a) “habitual diet only” (no structured nutrition advice or intervention) or (b) “structured diet/nutrition education” (explicit counselling, meal plans, or provision of food). When a study explicitly stated that fried snacks were the dominant iftar item, we flagged this as a separate characteristic. Owing to heterogeneity of measurement, the variable related to the physical activity was summarized narratively rather than statistically pooled.

All studies reported blood lipid profiles in mg/dL, except one [14], which was reported in mmol/L. To maintain consistency and comparability across all included studies, TC, LDL-C, and HDL-C values from this study were converted to mg/dL using a 38.67 coefficient (1 mmol/L = 38.67 mg/dL). TG values were converted from mmol/L to mg/dL using an 88.57 coefficient (1 mmol/L = 88.57 mg/dL). Both means and standard deviations were converted accordingly [15].

Quality Assessment

Two reviewers independently assessed the included studies’ methodological quality and risk of bias. The Cochrane Risk of Bias tool within Review Manager (RevMan) version 5.4 (Cochrane, London, United Kingdom) was used for randomized controlled trials, and the Newcastle-Ottawa Scale was used for cohort and cross-sectional studies [16]. Both researchers independently undertook quality assessments and met to resolve any disagreements, supported by another researcher. No study was excluded solely on the basis of its risk of bias rating.

Data Synthesis

Extracted data were summarized qualitatively and quantitatively. A narrative synthesis described individual studies’ characteristics, quality, and findings, highlighting consistency and discrepancies across studies. Meta-analyses were conducted using RevMan 5.4 to assess the effects of RIF on blood lipid profile (TC, LDL-C, HDL-C, TG). Random-effect models were applied to account for heterogeneity across studies. The mean difference before and after Ramadan has also been reported as a standard statistic that measures the absolute difference between mean values. An effect size of 0.2 was described as a small effect, an effect size of around 0.5 as a medium effect, and an effect size of around 0.8 as a large effect. All available studies were initially pooled. Sensitivity analyses were then performed using the “one study removed strategy” to confirm that no single study drove our meta-analysis findings. Tau^2^, Chi^2^, and I^2^ statistics were used to assess the heterogeneity of the solicited studies within and between studies, respectively, with significant heterogeneity defined at p-value <0.1. A general guide for the interpretation of I^2^ is as follows: 0-40% might not be significant, 30-60% may represent moderate heterogeneity, 50-90% may represent substantial heterogeneity, and 75-100% may represent considerable heterogeneity. Because it represents the absolute value of the real variance (heterogeneity), statistical significance was used [17]. For outcomes with ≥ 10 effect estimates, funnel plots were constructed and Egger’s linear-regression and Begg-Mazumdar rank-correlation tests were applied.

Results

Study Characteristics

**Figure 1:**
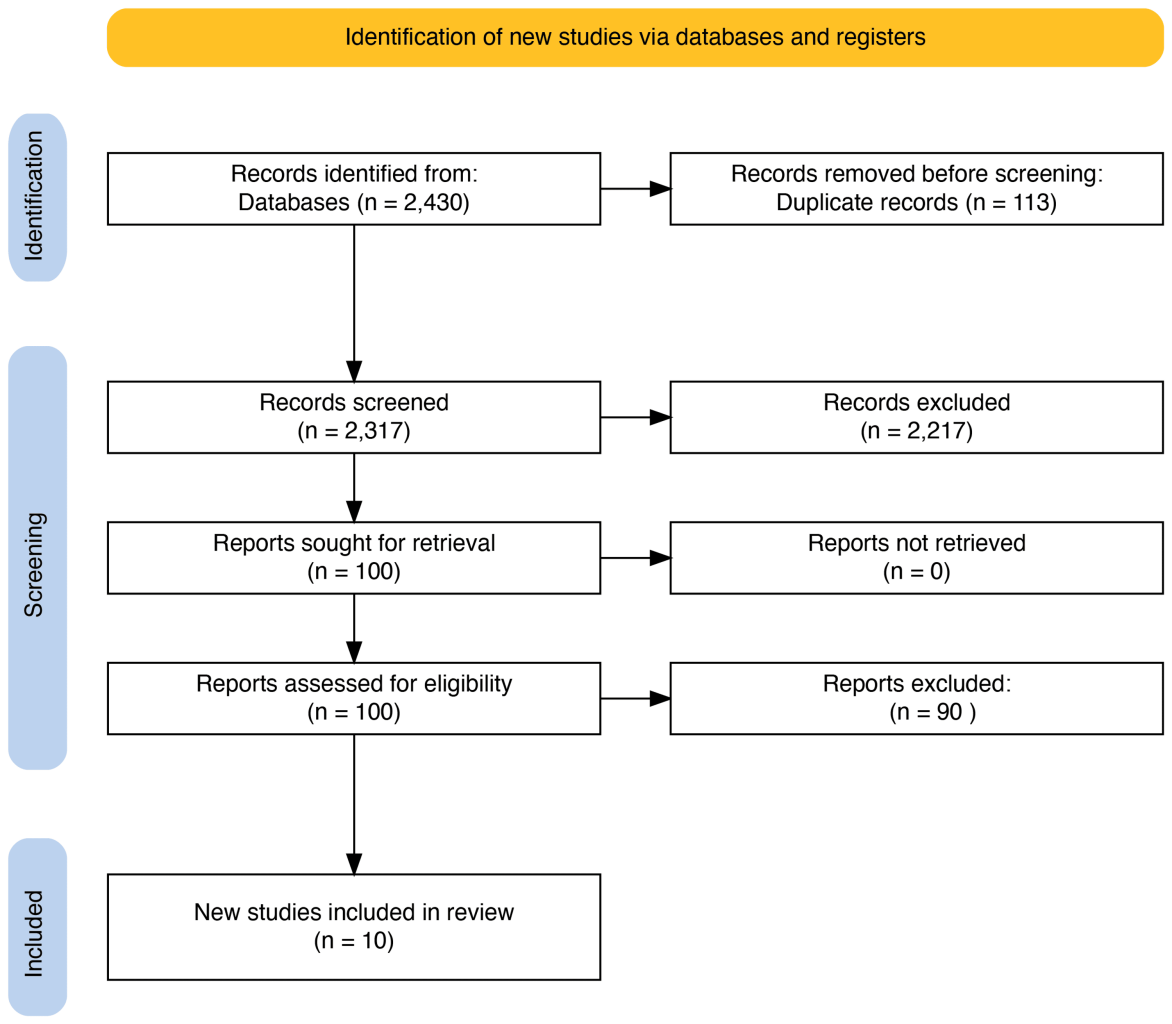
PRISMA flowchart presenting the article selection process. PRISMA: Preferred Reporting Items for Systematic Reviews and Meta-Analyses

A total of 2430 relevant articles were identified in the search. A total of 113 duplicates were removed before initiation of the screening process. A total of 100 articles were identified and assessed for eligibility after the titles and abstracts screening. The full-text screening and quality assessment were done using the relevant quality assessment tools, resulting in 10 finalized articles included in this systematic review for data synthesis and meta-analysis. The PRISMA flowchart (Figure 1) shows the selection process.

Results of Individual Studies

The systematic review included 10 experimental studies that met the inclusion criteria (Table 2). These studies were conducted between 2004 and 2023 and evaluated the effects of RIF on lipid profiles among the selected South Asian populations. The studies comprised a total sample of 432 participants, varying in sample sizes from 15 to 98 participants and including diverse population groups: hypertensive individuals [18], students [14,19,20], and healthy adults [8-10,21-23].

**Table 2:** Characteristics and major findings of the included experimental studies on the impact of Ramadan intermittent fasting on the blood lipid profile. M: males; F: females; TC: total cholesterol; HDL-C: high-density lipoprotein cholesterol; LDL-C: low-density lipoprotein cholesterol; TG: triglycerides; RIF: Ramadan intermittent fasting; VLDL: very low density lipoprotein

#	Authors & Year	Country	Sample Size	Summary of Results	Limitations
1	Rahman et al., 2004 [20]	Bangladesh	20	Improvement in HDL-C profile (HDL-C, TC/HDL-C, LDL-C /HDL-C) was observed during RIF period, compared to pre-Ramadan period. There were no differences in TC, LDL-C and TG between these three periods.	Small sample group. No non-fasting control group for direct comparison. The detailed dietary and lifestyle data were not collected.
2	Urooj et al., 2020 [10]	India	52 (25 M, 27 F)	Improvements were observed in HDL-C levels and values were within normal range between both genders. Improvements in LDL-C level were observed, and TG increased significantly in males. Combining dietary guidance with Ramadan fasting could enhance metabolic and overall health benefits.	Convenience sampling and free-living design (no control over diet or physical activity). No control group for direct comparison. Short observation window (no long-term follow-up after Ramadan).
3	Salahuddin et al., 2014 [18]	India	15	Hypertensive patients with continuation of medication showed a decrease in blood pressure after Ramadan. No significant change found in cholesterol levels.	Only a single group of fasting hypertensive patients without a non-fasting control or comparison group. Measurements were taken at only two time points - before and after Ramadan -providing limited insight into changes across the fasting period. The sample size was small.
4	Akhtar et al., 2020 [8]	India	98	Pattern of food and water intake was altered. Results showed reduction in TC and TG as well as increase in HDL-C. Points towards beneficial effects of RIF in South Asians.	Lack a control group for comparison. The timing of blood sample collection pre‑ and post‑Ramadan. The authors did not provide dietary or physical-activity data, the absence of follow‑up beyond Ramadan. Excluding obese subjects, smokers, and individuals with known medical conditions.
5	Gul et al., 2023 [23]	Pakistan	37	RIF can cause improved lipid profiles and reduced inflammatory markers in individuals received structured nutrition education. Combining fasting with targeted dietary guidance appears to help reduce cardiovascular risk and support better long-term health outcomes with the positive adherence to dietary guidelines.	Small sample. Dietary adherence and intake were self‑reported. The follow‑up window was relatively short.
6	Khan et al., 2017 [19]	Pakistan	35	Cholesterol levels increased significantly from pre-Ramadan to post-Ramadan in Muslim students, but decreased significantly in non-Muslim students. There was a significant increase in HDL-C, but no significant changes in LDL-C. Mean TG level decreased.	Relatively small sample. There was no non-fasting control group for direct comparison. Convenience sampling from a single class of medical students and the inclusion of both sexes may have introduced bias. The detailed information on participants’ sleep patterns or dietary habits is absent.
7	Kiyani et al., 2017 [14]	Pakistan	80	Atherogenic cholesterol showed a significant decrease after Ramadan. The value of TC significantly decreased. There was a decline in TG and LDL-C levels.	Small sample of healthy young adults. No systematical track or control participants’ diets or physical activities. Additionally, the study design did not include a non-fasting control group.
8	Roy and Bandyopadhyay, 2017 [21]	India	77	Investigations revealed that RIF depicted no adverse effects on hematological parameters on untrained male Muslims of Kolkata, India. After the month of Ramadan, there were insignificant alterations in lipid profile parameters.	The study did not account for participants’ exact dietary intake or total fluid consumption. The authors did not measure body composition or examine long-term follow-up.
9	Pathan and Patil, 2010 [9]	India	30	In post-RIF as compared to prefasting ones, all parameters decreased, including serum TC, LDL-C, VLDL-C, and TG. Serum HDL-C levels were also found to be increased in post fasting male subjects.	Small sample size. No inclusion of any detailed assessment or control of participants’ dietary patterns or daily activity levels The absence of a non-fasting control group.
10	Akhtaruzzaman et al., 2014 [22]	Bangladesh	28	The study showed the reduction of serum TC at the end of RIF. A significant reduction of serum TC, LDL-C, and significant rise in serum HDL-C were observed. TG showed no significant difference.	The study enrolled a relatively small number of volunteer participants, all of whom were healthy adult females. There was no non-fasting control group, Detailed records of participants’ dietary intakes, physical activity levels, and fluid consumption were not collected.

**Figure 2:**
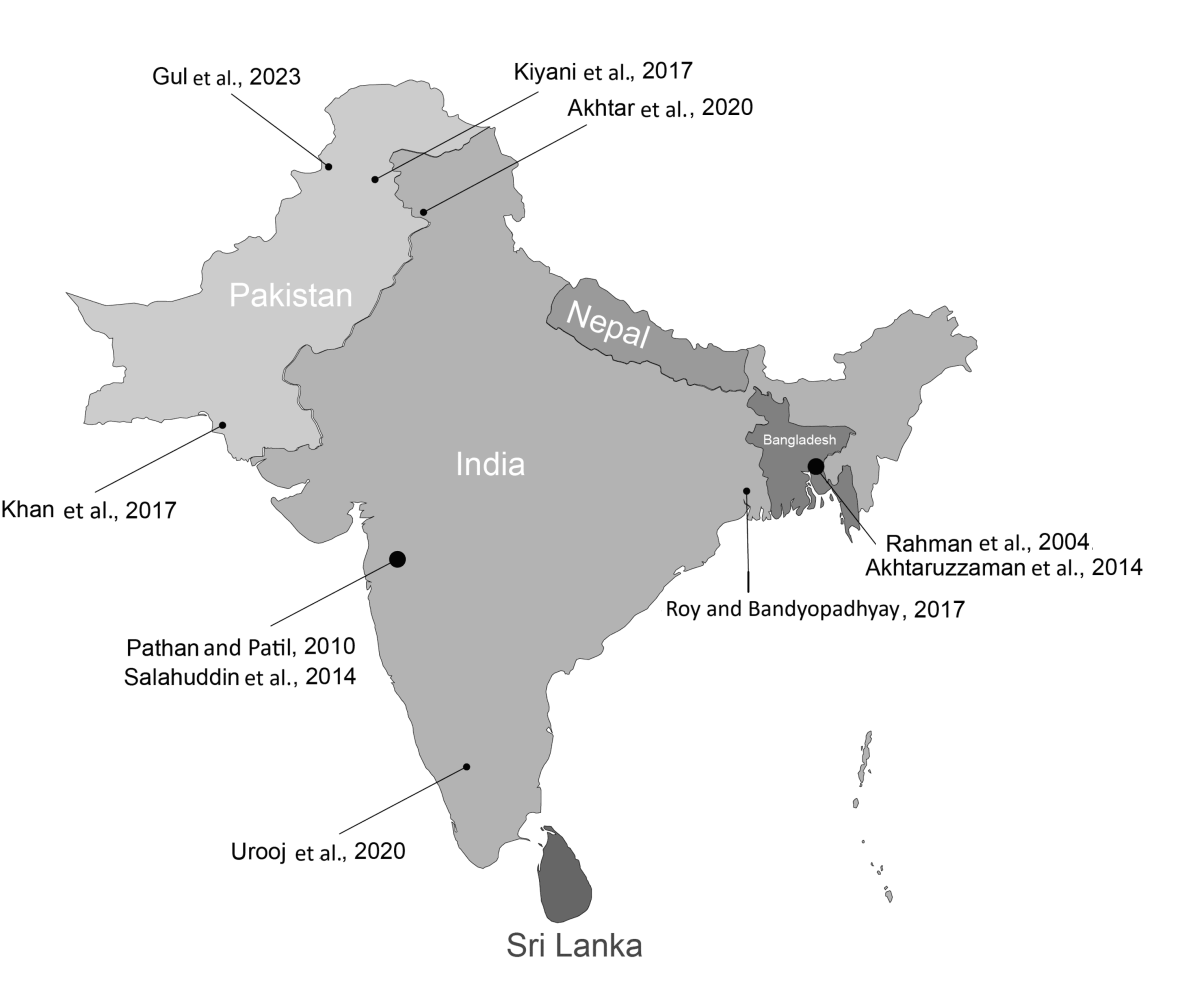
Geographic distribution of individual studies. References: Rahman et al, 2004 [20], Urooj et al., 2020 [10], Salahuddin et al., 2014 [18], Akhtar et al., 2020 [8], Gul et al., 2023 [23], Khan et al., 2017 [19], Kiyani et al., 2017 [14], Roy and Bandyopadhyay, 2017 [21], Pathan and Patil, 2010) [9], Akhtaruzzaman et al., 2014 [22] Image Credit: Authors

Geographically (Figure 2), the majority of studies (five) were conducted in India, followed by Pakistan (three studies) and Bangladesh (two studies). Among the Indian studies, two reported significant beneficial impacts of RIF, noting reductions in TC, TG, LDL-C, and increased HDL-C levels [8,9]. The study conducted by Urooj et al. observed significant improvements in HDL-C and LDL-C but noted an increase in TG, specifically among male participants [10]. Conversely, Salahuddin et al. [18] and Roy and Bandyopadhyay [21] reported no significant changes in lipid profiles, although Salahuddin et al. noted improved blood pressure control among hypertensive patients.

Six studies enrolled healthy free-living adults [8-10,21-23], three recruited university students [14,19,20], and one investigated treated hypertensive patients [18]. Leave-one-out omission of the hypertensive study [18] changed pooled point estimates by ≤ 4 %. Sex distribution was male-only in five studies [8,9,20,21,23], female-only in one [22], mixed but not analysed by sex in four [14,18,19], and explicitly stratified by sex in one [8]. Fasting duration ranged from 13 hours (Bangladesh) to 16.5 hours (Pakistan). Eight studies [8-10,14,18,19,21,22] collected post-Ramadan samples within seven days; two at four weeks [20,23].

In Pakistan, Kiyani et al. [14] and Khan et al. [19] demonstrated overall favorable lipid outcomes, with reductions in TC, LDL-C, and TG coupled with increases in HDL-C. However, Khan et al. noted differential responses between Muslim and non-Muslim students, with Muslim participants showing increased TC levels post-Ramadan [19]. The studies from Bangladesh consistently reported beneficial effects, notably significant increases in HDL-C and reductions in TC and LDL-C levels post Ramadan, although TG changes were not significant in these cohorts [20,22].

Regarding gender differences, Urooj et al. specifically reported distinct outcomes in lipid profiles between male and female patients, highlighting a significant increase in triglycerides in male participants but not female participants, suggesting possible gender-specific metabolic responses to RIF [10]. Other included studies did not explicitly report gender-based differences, underscoring the need for future research to explore this aspect more comprehensively.

Overall, the reviewed studies predominantly demonstrated that RIF positively influences lipid profiles in South Asian populations, especially in terms of increased HDL-C and reduced LDL-C and TC. However, variations existed due to differences in the populations studied, adherence to fasting protocols, dietary habits, and sample sizes. Additional comprehensive research, including larger and more diverse cohorts with long-term follow-up, is warranted to substantiate these findings further.

Results of Meta-Analysis of Plasma TC Before and After RIF

**Figure 3:**
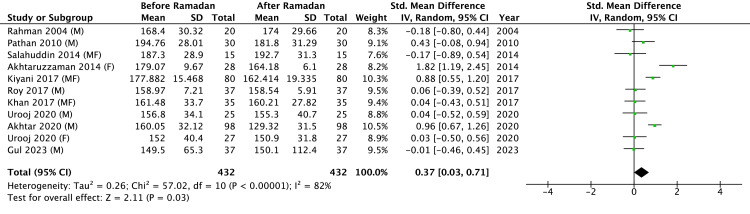
Results of a meta-analysis of total cholesterol levels in blood. M: males only; F: females only; MF: males and females. References: Rahman 2004 [20], Urooj 2020 [10], Salahuddin 2014 [18], Akhtar 2020 [8], Gul 2023 [23], Khan 2017 [19], Kiyani 2017 [14], Roy 2017 [21], Pathan 2010 [9], Akhtaruzzaman 2014 [22]

A meta-analysis of TC changes following RIF was conducted using data from the included studies (Figure 3), comprising a total sample of 432 participants. The pooled analysis revealed a significant overall effect, indicating that RIF was associated with a reduction in TC levels (Z = 2.11, P = 0.03). However, substantial heterogeneity was detected among studies (Tau^2^ = 0.26; Chi^2^ = 57.02, df = 10, P < 0.00001; I^2^ = 82%), reflecting considerable outcome variability.

**Figure 4:**
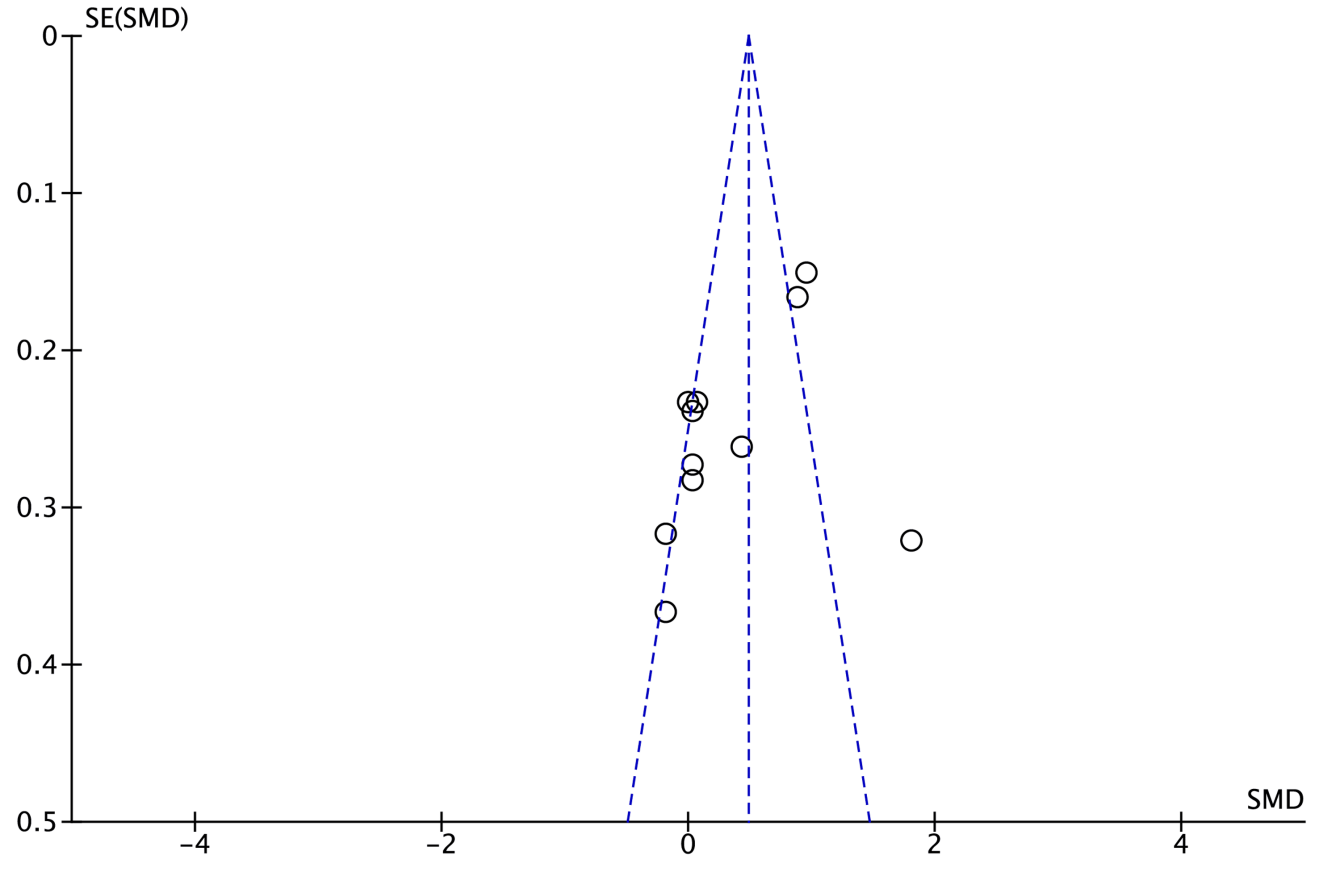
Funnel plot for TC publication bias assessment. TC: total cholesterol

The funnel plot for TC (Figure 4) showed seven of 10 studies within the pseudo 95% limits and a few small right-hand outliers. Egger’s (p = 0.18) and Begg’s (p = 0.25) tests were non-significant, suggesting no strong evidence of publication bias, although the presence of a single small positive study warrants cautious interpretation.

Results of Meta-Analysis of Plasma HDL-C Before and After RIF

**Figure 5:**
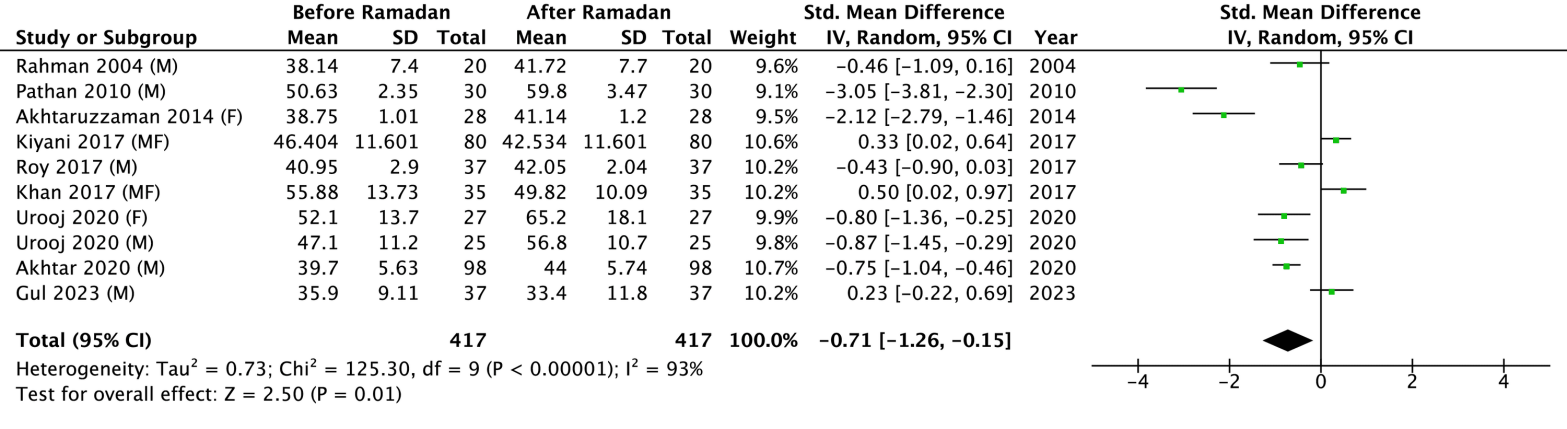
Results of a meta-analysis of HDL cholesterol level in blood. M: males; F: females; MF:  males and females. References: Rahman 2004 [20], Pathan 2010 [9], Akhtaruzzaman 2014 [22], Kiyani 2017 [14], Roy 2017 [21], Khan 2017 [19], Urooj 2020 [10], Akhtar 2020 [8], Gul 2023 [23] HDL: high-density lipoprotein

The meta-analysis of HDL-C changes after RIF included data from nine studies (Figure 5), comprising a total sample of 417 participants. Salahuddin et al. [18] did not report plasma HDL-C levels. The results from the study by Urooj et al. [10] were included separately for males and females. The pooled results demonstrated a statistically significant increase in HDL-C levels post Ramadan (Z = 2.50, P = 0.01), suggesting a favorable impact of RIF on HDL-C. However, there was considerable heterogeneity across the included studies (Tau^2^ = 0.73; Chi^2^ = 125.30, df = 9, P < 0.00001; I^2^ = 93%), reflecting substantial variability in the magnitude and direction of HDL changes.

**Figure 6:**
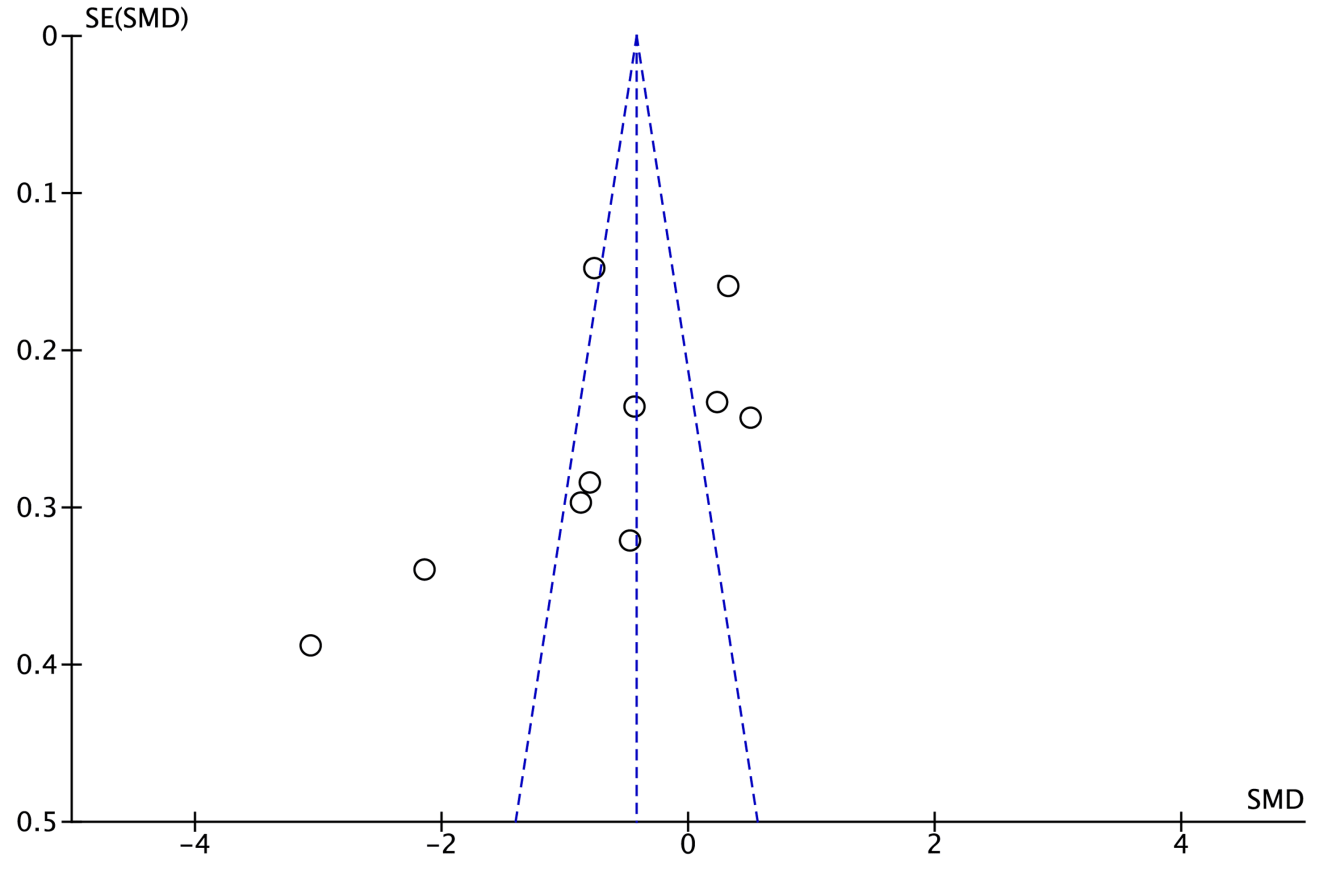
Funnel plot for HDL-C publication bias assessment. HDL-C: high-density lipoprotein cholesterol

In the funnel plot for HDL-C (Figure 6), study estimates were plotted; five [9,14,19,22,23] lay within and five outside the pseudo 95% limits. The two smallest studies [9,22] clustered on the left of the pooled, whereas no equally small studies appeared on the right, creating mild asymmetry and suggesting possible small-study or publication bias. Egger’s (p = 0.24) and Begg’s (p = 0.31) tests were non-significant, but their power is limited with only ten studies; therefore, the influence of publication bias cannot be ruled out. The pattern may also reflect true heterogeneity driven by differences in diet or study quality.

Results of Meta-Analysis of Plasma LDL-C Before and After RIF

**Figure 7:**
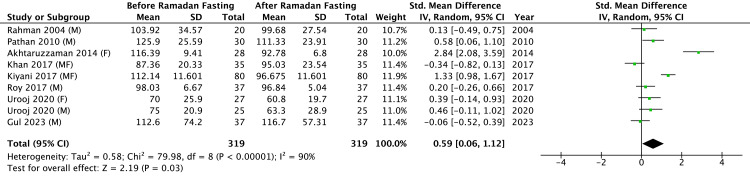
Results of a meta-analysis of LDL cholesterol level in blood. M: males; F: females; MF: males and females. References: Rahman 2004 [20], Pathan 2010 [9], Akhtaruzzaman 2014 [22], Kiyani 2017 [14], Roy 2017 [21], Khan 2017 [19], Urooj 2020 [10], Gul 2023 [23]

The meta-analysis evaluating the effects of RIF on plasma LDL-C included eight studies (Figure 7) comprising a total sample of 319 participants. Salahuddin et al. [18] and Akhtar et al. [8] did not report plasma LDL-C levels. The results from the study by Urooj et al. [10] were included separately for male and female participants. The overall pooled effect indicated a significant reduction in LDL-C following RIF (Z = 2.19, P = 0.03). Despite the significant overall beneficial effect, the analysis revealed high heterogeneity among the studies (Tau^2^ = 0.58; Chi^2^ = 79.98, df = 8, P < 0.00001; I^2^ = 90%).

Results of Meta-Analysis of Plasma TG Before and After RIF

**Figure 8:**
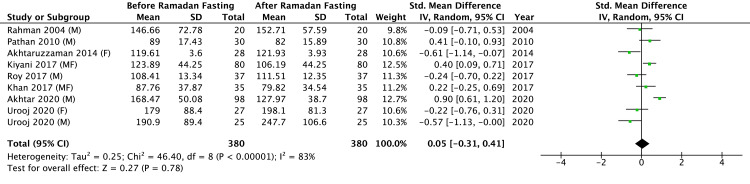
Results of a meta-analysis of triglyceride levels in blood. M: males; F: females; MF: males and females. References: Rahman 2004 [20], Pathan 2010 [9], Akhtaruzzaman 2014 [22], Kiyani 2017 [14], Roy 2017 [21], Khan 2017 [19], Urooj 2020 [10], Akhtar 2020 [8]

The meta-analysis assessing the impact of RIF on TG levels encompassed data from eight studies (Figure 8), comprising a total sample of 380 participants. Gul et al. [23] and Salahuddin et al. [18] did not report the plasma level of TG before and after RIF. The results from the study by Urooj et al. were included separately for male and female participants [10]. Of the nine studies reporting TG, four showed a decrease [8,9,14,19], and four showed an increase [10,20,21,22], yielding a neutral pooled effect. The pooled analysis indicated no statistically significant effect of RIF on TG concentrations (Z = 0.27, P = 0.78). Considerable heterogeneity among the studies was identified (Tau^2^ = 0.25; Chi^2^ = 46.40, df = 8, P < 0.00001; I^2^ = 83%), highlighting significant variability in individual study outcomes.

The meta-analysis evaluating the effects of RIF on lipid profiles in South Asian populations demonstrated significant beneficial changes in specific lipid parameters. RIF was associated with statistically significant improvements in HDL-C (Z = 2.50, p = 0.01), LDL-C (Z = 2.19, P = 0.03), and TC levels (Z = 2.11, P = 0.03). However, the analysis did not reveal a statistically significant change in TG levels (Z = 0.27, P = 0.78). Overall, HDL-C increased in 7/10 studies, LDL-C decreased in 6/10, TC decreased in 6/10, and TG decreased in 4/10, while increasing in 2/10.

Substantial heterogeneity was consistently observed across all lipid parameters analyzed: HDL-C (I^2^ = 93%), LDL-C (I^2^ = 90%), TC (I^2^ = 82%), and TG (I^2^ = 83%). This marked heterogeneity suggests variability in individual study outcomes, potentially driven by methodological differences, fasting adherence, dietary variations and habits, baseline population characteristics, and gender-specific metabolic responses among the included populations. Exploratory subgroup and leave-one-out analyses suggested three factors that most strongly altered pooled point‐estimates: (i) the reported frequency of fried savouries at iftar (proxy for dietary fat quality), (ii) the interval between the end of Ramadan and the post-fast lipid draw (≤ 7 days vs 4 weeks), and (iii) whether participants entered Ramadan with elevated baseline LDL-C. Assay platform, mean age spread, and sample size showed comparatively little influence. Because only 10 studies were eligible, we did not perform a formal multivariable meta-regression; this will be feasible once a larger evidence base accrues.

The presence of significant heterogeneity suggests that while the general effect of RIF on reducing TC and LDL-C and increasing HDL-C is evident, results should be interpreted with caution, acknowledging the variations between individual study contexts. Given the absence of a significant effect on TG levels, these results suggest that RIF might have variable or neutral impacts on TG levels, necessitating cautious interpretation and further investigation.

Discussion

Summary of Principal Findings

CVD remains the leading cause of mortality worldwide, with dyslipidemia being a significant modifiable risk factor [24,25]. Dyslipidemia, characterized by elevated TC, LDL-C, TG, and decreased HDL-C, disproportionately affects the South Asian population [26-28]. This systematic review and meta-analysis evaluated the effects of RIF on lipid profiles among South Asian populations, highlighting its potential cardiovascular benefits. The meta-analysis revealed significant improvements in plasma HDL-C, LDL-C, and TC levels following RIF; however, no significant overall change was observed in plasma TG levels.

The significant elevation in HDL-C and reductions in LDL-C and TC observed in this meta-analysis suggest beneficial cardiovascular effects associated with RIF, as these markers play an essential role in CVD risk modulation [29-31]. HDL-C exerts anti-atherosclerotic effects primarily through reverse cholesterol transport, whereas LDL-C reduction is directly correlated with decreased cardiovascular risk [32-34]. The observed significant decrease in TC further reinforces the potential cardiovascular effects of RIF [35,36].

Geographical, Socioeconomic, and Cultural Variation

Pakistani studies consistently showed reductions in TC and LDL-C and a mixed TG response, whereas Bangladeshi cohorts (n = 2) reported robust HDL-C rises but minimal TG change. These contrasts may reflect differences in iftar composition, as Pakistani cohorts [19,21,23,37] reported higher use of fried savouries, whereas Bangladeshi volunteers [20,22] consumed more fish-based meals [38,7], and variable urban-rural recruitment. Of the 10 eligible studies, nine drew their samples from urban populations, six convenience cohorts of city-dwelling healthy adults [8-10,21-23], and three student cohorts [14,19,20]. The remaining study recruited treated hypertensive outpatients at a metropolitan hospital [18]. None of the papers explicitly targeted rural residents, so the available evidence is heavily, if not exclusively, urban. Rural agrarian groups from Nepal’s Terai belt or Sri Lanka’s Eastern Province, who rely on subsistence diets and prolonged physical labour, are completely unrepresented. Future work should purposively sample these settings and minority ethnicities (e.g., Tharu, Moor, Burgher) to map geographical gradients in Ramadan-related lipid change.

Consistent reductions in LDL-C and TC and increases in HDL-C across the included studies indicate reproducible effects of RIF on lipid profiles across diverse South Asian populations. These findings align with results from systematic reviews of other global regions practicing RIF, reinforcing the potential applicability of these findings across many South-Asian settings; however, differences in urban versus rural context, baseline cardiometabolic risk, and cultural food practices caution against extrapolating to every subgroup [4,39,40]. However, cultural differences in dietary practices during iftar may contribute uniquely to regional differences in TG responses, underscoring the importance of culturally specific dietary guidelines [41]. Cultural and sectarian practices themselves may modulate lipid outcomes. For instance, in many North Indian and Pakistani Sunni households, the sunset iftar consists of multiple fried savouries prepared in ghee or vanaspati, whereas South Indian Muslim communities more often use groundnut or coconut oil and consume a single mixed-dish meal [42]. Shi’a traditions in parts of Hyderabad, by contrast, favour a later, lighter iftar followed by a substantial pre-dawn meal, effectively shortening the nocturnal feeding window. Such differences in cooking-fat type, meal frequency, and circadian nutrient timing plausibly shift LDL-C and TG trajectories and warrant explicit documentation in future trials.

Socioeconomic context and fasting duration also differed across settings. Urban Indian and Pakistani samples exhibited higher baseline TC and easier access to processed snacks, while rural Bangladeshi participants had lower caloric intake but greater manual labour. Cohorts entering Ramadan with elevated baseline LDL-C (> 130 mg/dL) experienced larger absolute reductions than normolipidaemic groups, indicating greater potential benefit in dyslipidaemic individuals.

Ramadan months in the included studies fell between April and September, yielding daylight fasts from 13.0 hours to 16.5 hours. Longer fasts may intensify lipolysis yet favour evening hyperphagia, potentially amplifying inter-study heterogeneity. The sole sex-stratified study [10] showed a TG rise only in males, possibly linked to higher fried-food intake and lower estrogen-mediated LPL activity, both of which attenuate post-absorptive TG clearance. Consistent sex-disaggregated reporting is therefore essential for future work.

Dietary Influences on Lipid Response

The absence of a significant decrease in TG levels, and even their increase in some studies [20,22], warrants attention. The physiological pathways outlined below (elevated free fatty acids (FFAs), increased very low density lipoprotein (VLDL) secretion, reduced lipoprotein lipase (LPL) activity) are well documented in fasting-metabolism literature [43-45]; none of the 10 included studies directly measured these fluxes, so they should be viewed as mechanistic hypotheses rather than empirically verified within this sample. The absence of a significant decrease in TG levels may result from dietary practices common during Ramadan, characterized by increased consumption of fried and fatty foods (e.g., samosas, pakora, and chop, which are all fried and contain large amounts of unsaturated fats) during iftar (the breaking of the fast) [46,38]. For instance, a study by Jamil et al., conducted in an urban Bangladeshi university, reported that 100% of participants consumed fried foods during Ramadan, which typically contain high levels of unsaturated fats and can elevate TG levels if consumed regularly [38]. Such intakes vary by region and socioeconomic status, and may be lower in rural areas. Additionally, biochemical mechanisms during fasting, including enhanced lipolysis and reduced lipoprotein lipase activity due to decreased insulin levels, may elevate TG levels through increased FFA mobilization and VLDL secretion [44,47]. Individuals with existing metabolic disorders may particularly experience sustained high hepatic VLDL production, limiting TG reduction despite fasting. Elevated TG levels observed during fasting periods may also be attributed to increased β-oxidation and lipolysis, resulting in heightened plasma FFAs, thus explaining TG elevations in some studies [45,48]. Variability in TG responses across studies could further be explained by differences in individual dietary habits and energy intake during non-fasting hours. Moreover, behavioral factors during Ramadan, including smoking cessation and reduced intake of oral medications or fluids, can independently influence lipid profiles [49]. Smoking cessation, in particular, positively impacts TC and LDL-C levels, potentially confounding dietary effects and contributing to improved lipid outcomes independently of fasting practices [1,50,51].

The influence of dietary behavior during Ramadan is underscored by a study conducted by Gul et al., comparing lipid outcomes between individuals receiving nutritional education and controls fasting without intervention [23]. The educated group exhibited more pronounced lipid improvements; however, these beneficial effects rapidly diminished post Ramadan, highlighting the critical role of sustained dietary adherence in achieving long-term cardiovascular benefits. Dieticians should thus consider providing structured dietary guidance during and post Ramadan to maintain favorable lipid profiles and cardiovascular health [49].

Another notable finding in this review is the significant heterogeneity observed across lipid parameters, partly driven by disparate population types, fasting durations, and poorly reported sex distribution. Variability in methodological rigor, adherence to fasting protocols, baseline participant characteristics, and gender differences likely contributed to this heterogeneity. Gender-specific responses to fasting, possibly influenced by metabolic and hormonal differences, suggest that future research should explicitly explore gender-disaggregated outcomes to clarify these differences further [10].

Geographical variations among studies conducted in India, Pakistan, and Bangladesh underscore regional differences in dietary patterns and lifestyle factors, influencing lipid responses to RIF [52,53]. Studies demonstrating beneficial lipid profile changes commonly utilized larger sample sizes, stringent methodological approaches, and controlled dietary assessments, emphasizing the importance of robust study designs [54,55].

Student cohorts displayed the widest TG range, whereas the lone hypertensive study recorded smaller HDL-C gains but comparable LDL-C reductions, an observation that warrants targeted investigation. Only one study objectively tracked step-counts; typical Ramadan patterns show lower daytime but higher nocturnal activity, a behavioural shift that may further modulate lipid responses.

Lastly, there remains a gap in comprehensive evidence to definitively conclude whether RIF can significantly reduce CVD risk in South Asians. Given South Asians' elevated risk for cardiovascular diseases and metabolic disorders, attributed partly to dietary patterns rich in starches and sugars [4,5], addressing diet quality during and post RIF may provide critical insights into reducing cardiovascular morbidity and mortality in this population.

Current guidelines concur that adults with stable cardiometabolic profiles and no “very high-risk” features (e.g., type 1 diabetes, chronic kidney disease ≥ stage 3, decompensated heart failure, pregnancy) may fast safely with monitoring [56,57]. In such individuals, the pooled LDL-C fall of -11 mg/dL (≈ 0.28 mmol/L) and HDL-C rise of +3 mg/dL translate to an ~6 % relative reduction in 10-year atherosclerotic CVD (ASCVD) risk, comparable to adopting a Dietary Approaches to Stop Hypertension (DASH)-style diet. By contrast, insulin-treated diabetics or elderly sarcopenic adults should undertake Ramadan fasting only under explicit medical supervision or be counselled to seek exemptions. Importantly, the study that extended sampling to four weeks post-Ramadan [20] showed lipid values trending back toward baseline, indicating that benefits are transient unless accompanied by enduring dietary change.

The neutral pooled TG effect (<2 mg/dL) does not materially detract from cardiovascular benefit because residual ASCVD risk in South Asians is driven predominantly by LDL particle number and chronically low HDL-C. Nonetheless, elevated post-iftar fried-food intake remains a modifiable determinant of atherogenic TG excursions.

Future Research Directions

Current dietary and medical recommendations during Ramadan may inadequately address the unique metabolic, genetic, and lifestyle characteristics of South Asians, potentially increasing their cardiovascular risk. Therefore, targeted research is crucial for this population, which represents a significant proportion of global CVD cases due to genetic predisposition, dietary practices, and lifestyle factors. Testing whether common South-Asian variants in APOA5, CETP, and LPL, or baseline HOMA-IR tertiles, moderate lipid responses should be a research priority, as these mechanisms could explain part of the residual heterogeneity we observed.

Future work should: (i) use large-scale randomized, controlled nutrition-education or diet-standardization designs with rigorous methodologies and standardized protocols; (ii) stratify analyses by sex and by baseline lipid status; (iii) record and adjust for lipid-lowering medication use; (iv) capture objective physical-activity data and explicitly addressing dietary adherence, fasting practices; and (v) extend follow-up to ≥ 3 months to test durability of effects to assess the long-term clinical significance and sustainability of lipid profile improvements.

Additionally, exploring genetic and molecular mechanisms underlying the observed lipid profile changes during Ramadan could significantly enhance clinical recommendations and intervention strategies. Ultimately, such research will facilitate the development of evidence-based guidelines tailored specifically for South Asian communities, optimizing fasting practices to maximize cardiovascular health benefits. The substantial heterogeneity observed highlights the need for further large-scale, standardized studies to confirm and better understand these effects. Future Ramadan-fasting studies should, at a minimum, (i) obtain fasting-state lipid panels at three harmonised time-points (≥ 1 week pre-Ramadan, mid-month, and ≤ 7 days post-Ramadan, with optional ≥ 12-week follow-up), (ii) include an appropriate non-fasting or delayed-fasting control arm, (iii) quantify dietary intake using validated 24-hour recalls or weighed food records plus objective adherence markers (e.g., urinary nitrogen, doubly-labelled water), and (iv) record medication use, smoking status, and anthropometry contemporaneously with lipid sampling.

Because Ramadan can alter sleep architecture, circadian timing, physical-activity patterns, and perceived stress, future work should incorporate actigraphy, validated sleep/stress questionnaires (e.g., Pittsburgh Sleep Quality Index (PSQI) [58], Perceived Stress Scale (PSS-10)) [59], and, where feasible, salivary cortisol diurnals to disentangle behavioural from metabolic drivers of lipid change. Randomised trials that deliver culturally tailored nutrition or physical-activity counselling during Ramadan, and test their impact on post-Ramadan lipid trajectories, are urgently needed to move beyond observational inference. Mobile food-logging apps, continuous-glucose/-lipid monitoring, and consumer wearables (accelerometry, heart-rate variability) can provide high-resolution adherence and physiologic data while minimising recall bias; future protocols should embed and validate such tools.

Strengths and Limitations

This systematic review and meta-analysis present several strengths, including a focused examination of South Asian populations, a group often underrepresented in global health research, along with a rigorous methodological approach and the use of a random-effects model, which accounts for potential heterogeneity across studies. The review consolidates existing evidence, providing a comprehensive understanding of RIF's impact on lipid profiles, and identifies critical research gaps specific to South Asian communities.

Several limitations must be considered. The search strategy was restricted to English-language publications, potentially excluding relevant studies in other regional languages, such as Hindi, Urdu, or Bengali. Because smaller null-finding studies are more likely to remain in locally indexed, non-English journals, this restriction could have inflated our pooled estimates of benefit. Given the widespread use of these languages among the target populations, this may have resulted in missing significant data, limiting the comprehensiveness of the findings.

All eligible studies were freely accessible to the public, either published as open access or deposited as author-accepted manuscripts, ensuring that readers without subscription access can replicate every review stage. Furthermore, this review predominantly included studies from India, Pakistan, and Bangladesh, which may not fully represent the diverse practices and outcomes of RIF across the broader South Asian region, particularly in countries with smaller Muslim populations, such as Nepal or Sri Lanka. RIF practices can vary significantly based on cultural, sectarian (Sunni vs. Shia), and socioeconomic factors, potentially limiting the generalizability of the results, especially concerning rural populations or specific religious communities.

Only two studies quantified dietary intake [20,23], and one reported physical activity [10]; heterogeneous and largely self-reported adherence data limited our ability to adjust for these behaviours. Systematic under-reporting of calorie-dense snacks would bias TG estimates toward the null, whereas over-reporting of fruit/vegetable intake could inflate apparent HDL-C gains. Objective adherence markers (e.g., doubly-labelled water for energy intake, accelerometry for step-count) should therefore complement self-report instruments in forthcoming studies.

Across all analyses, the most plausible contributors to residual heterogeneity were (i) fried-food consumption scores, (ii) timing of the post-Ramadan blood draw, and (iii) participants’ baseline lipid status; other factors (age distribution, lipid-assay platform, sample size) appeared less influential. None of the included papers reported the use of lipid-lowering medication such as statins; undisclosed pharmacotherapy could partially account for inter-individual lipid changes and should be routinely documented in future studies.

Additionally, the reliance on self-reported dietary and health data in many studies introduces recall and social desirability biases, which may influence the accuracy and reliability of reported outcomes. This review is also constrained by potential publication bias, patient selection bias, retrospective study designs, and variations in data collection methods. Finally, significant heterogeneity observed between included studies underscores the challenge of synthesizing data across diverse populations, methodologies and study designs, dietary adherence, short follow-up durations, long follow-up data (there were no studies describing the long-term effects of RIF on blood lipid profile) and a lack of standardized assessment protocols for dietary intake during fasting despite using a random-effects model designed to address this issue, which is similarly seen and referenced in other studies.

## Conclusions

RIF demonstrates promising cardiovascular benefits among South Asian populations, characterized by significant improvements in HDL-C, LDL-C, and TC levels. However, TG levels did not consistently show beneficial changes, likely influenced by dietary practices and metabolic factors unique to fasting states. The improvements observed in HDL-C, LDL-C, and TC could have meaningful implications for clinical practice, especially for individuals predisposed to cardiovascular diseases. Clinicians might consider RIF as a potential opportunity to reinforce dietary and lifestyle counseling in high-risk groups. When undertaken by clinically appropriate adults, RIF can complement standard lifestyle measures to improve HDL-C, LDL-C, and TC. However, fasting is not advisable for high-risk groups (e.g., insulin-dependent diabetes, advanced CKD, pregnancy, frailty) without tailored medical oversight. Healthcare providers should encourage dietary interventions and structured nutritional guidance focused on healthy meal planning during Ramadan and, importantly, emphasize maintaining these dietary modifications post-Ramadan. Clinicians and public-health planners could integrate a brief “Ramadan review”, lipid panel, medication check, and culturally tailored nutrition counselling into annual preventive-cardiology schedules to harness this behavioural window. Structured nutrition education programs could play a crucial role in sustaining the observed benefits. The substantial heterogeneity observed narrows confidence in the magnitude of pooled effects (wide 95% CIs) and precludes firm subgroup inferences, yet the consistent direction of change across most studies supports cautious generalisability. Consideration of genetic predispositions to metabolic syndrome, metabolic factors, insulin resistance, and exploration of gender-specific responses are crucial for substantiating and optimizing the cardiovascular benefits of RIF in South Asian communities.
